# NK cell development requires Tsc1-dependent negative regulation of IL-15-triggered mTORC1 activation

**DOI:** 10.1038/ncomms12730

**Published:** 2016-09-07

**Authors:** Meixiang Yang, Shasha Chen, Juan Du, Junming He, Yuande Wang, Zehua Li, Guangao Liu, Wanwen Peng, Xiaokang Zeng, Dan Li, Panglian Xu, Wei Guo, Zai Chang, Song Wang, Zhigang Tian, Zhongjun Dong

**Affiliations:** 1 Institute for Immunology and School of Medicine, Tsinghua University, Beijing 100086, China; 2 Biomedical Translational Research Institute, Jinan University, Guangzhou 510632, China; 3 School of Medicine, Tsinghua University, Beijing 100086, China; 4 Center of Animal Facility, Tsinghua University, Beijing 100086, China; 5 Collaborative Innovation Center, Wuhan Sports University, Wuhan 340036, China; 6 School of Life Sciences, University of Sciences and Technology of China, Hefei 230026, China

## Abstract

Activation of metabolic signalling by IL-15 is required for natural killer (NK) cell development. Here we show that Tsc1, a repressor of mTOR, is dispensable for the terminal maturation, survival and function of NK cells but is critical to restrict exhaustive proliferation of immature NK cells and activation downstream of IL-15 during NK cell development. *Tsc1* is expressed in immature NK cells and is upregulated by IL-15. Haematopoietic-specific deletion of *Tsc1* causes a marked decrease in the number of NK cells and compromises rejection of ‘missing-self' haematopoietic tumours and allogeneic bone marrow. The residual *Tsc1*-null NK cells display activated, pro-apoptotic phenotype and elevated mTORC1 activity. Deletion of *Raptor*, a component of mTORC1, largely reverses these defects. *Tsc1*-deficient NK cells express increased levels of T-bet and downregulate Eomes and CD122, a subunit of IL-15 receptor. These results reveal a role for Tsc1-dependent inhibition of mTORC1 activation during immature NK cell development.

## Abstract (web summary)

IL-15 orchestrates NK cell metabolism, proliferation, and activation. Here the authors show that Tsc1 is dispensable for mature NK cells but is critical for survival of immature NK by preventing their exhaustive proliferation and activation downstream of IL-15.

## Graphical abstract

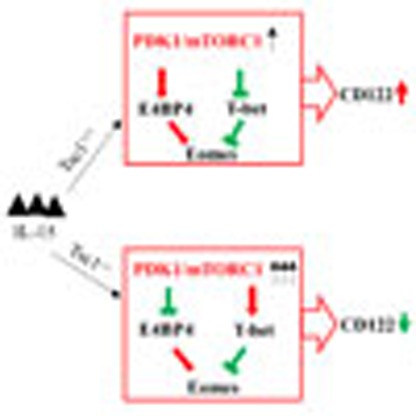

Natural killer (NK) cells have critical roles in innate immunity against ‘unwanted' cells, such as transformed tumour cells and virally infected cells, as well as that against class I major histocompatibility complex (MHC-I)-mismatched allogeneic bone marrow. NK cell development occurs in the bone marrow of adults, and it is strictly dependent on signalling triggered by the pleiotropic cytokine interleukin-15 (IL-15). Thus, mice lacking either IL-15 or any one of the IL-15 receptor subunits, including the α, β and γ chains, display severely abnormal NK cell development^1^,^2^. IL-15 signalling is also thought to be involved in the maintenance of NK cell survival and homeostasis in peripheral immune niches. This cytokine supports NK cell development and survival mainly through the activation of a FOXO-dependent transcriptional programme and the prevention of Bim-mediated apoptosis^3^,^4^,^5^. IL-15-mediated signalling is also essential for NK cell homeostasis *in vivo*, primarily due to its regulation of the level of the anti-apoptotic protein Mcl-1 (ref. 6). In view of its importance to NK cell physiology, IL-15 signalling must be actively regulated to prevent its aberrant activation, which is likely detrimental to NK cell development, homeostasis and quiescence.

IL-15-responsive NK cell progenitors (NKp), characterized by the expression of IL-2Rβ (CD122), a subunit of IL-15 receptor, require the common gamma chain (γc/IL-2Rγ/CD132) to transduce activating signals. IL-15 stimulation primarily activates the kinase function of Janus kinase (JAK), which leads to phosphorylation and activation of signal transducer and activator of transcription 3 (STAT3) and STAT5. Thus, humans or mice lacking JAK and STAT5A/B display impaired NK cell development^7^,^8^. IL-15 dramatically induces the expression of suppressor of cytokine signalling 2 (SOCS2) during its late phase of stimulation, which negatively modulates the JAK-STAT pathway, suggesting that SOCS2 is likely required for NK cell function due to its downregulation of IL-15 signalling^9^.

IL-15 also has the ability to biochemically activate PI3K pathways^10^. Mice simultaneously lacking the PI3K subunits P110 γ and δ have severe defects in early NK cell development^11^,^12^. Most recently, we have observed that PDK1 deficiency causes an almost 95% reduction in the number of NK cells^13^, comparable to p110 γ and δ double-mutant mice^14^ strongly suggesting that PI3K regulates NK cell development largely via the recruitment of PDK1. PI3K activity is negatively controlled by phosphatase and tensin homologue deleted on chromosome ten and Src homology 2 domain-containing inositol-5-phosphatase-1. Mice lacking either phosphatase and tensin homologue deleted on chromosome 10 or Src homology 2 domain-containing inositol-5-phosphatase-1 exhibit abnormal NK cell development and functionality, demonstrating the crucial roles of these two proteins in actively maintaining NK cell function^15^,^16^,^17^.

The distal signalling downstream of PI3K activation activates mammalian target of rapamycin (mTOR), which is a component of two active complexes, mTOR complex 1 (mTORC1) and mTORC2. mTOR kinase has been reported to play a crucial role as a key metabolic checkpoint in NK cell proliferation and activation^18^. The signalling mediated by mTOR regulates early NK cell development mainly via the induction of E4BP4, an NK cell-specific transcription factor, which maintains IL-15 responsiveness by upregulating CD122 expression. Therefore, the activity of IL-15 is amplified via a positive feedback loop^13^; however, the activation of mTOR must be tightly regulated according to this activity. One of the most indispensable proteins involved in this process is tuberous sclerosis 1 (Tsc1). This peripheral membrane protein has been implicated as a tumour suppressor. Tsc1 forms a complex with Tsc2, which regulates mTORC1 signalling, and this process is mediated by Akt. Human *Tsc1* mutations may result in tuberous sclerosis by causing functional impairment of the hamartin-tuberin complex. Interestingly, Tsc1 plays critical roles in immune processes, such as T-cell differentiation^19^,^20^, peripheral T-cell homeostasis^21^, dendritic cell development^22^ and natural killer T (NKT) cell terminal differentiation^23^. Tsc1 is also required for the generation of memory CD8^+^ cells, a process that strictly requires IL-15 signalling^24^. However, it remains unknown whether Tsc1 is necessary for restraining IL-15/mTOR signalling during NK cell development, homeostasis and functioning.

To address how IL-15 signalling is negatively regulated in NK cells, in the current study, we examine dynamic changes in the expression of negative regulators of two of the above-mentioned signalling pathways, JAK-STAT and PI3K-mTOR, after IL-15 triggering. *Tsc1* is found to be upregulated at the late time point of IL-15 stimulation. Thus, we generate *Tsc1*-deficient mice to dissect the roles of Tsc1 in IL-15 signalling and NK cell development. We identify a unique role of Tsc1-dependent inhibition of mTORC1 activity in the optimization of IL-15 signalling in the early stage of NK cell development.

## Results

### IL-15 stimulation upregulates Tsc1 in NK cells

**Figure 1:**
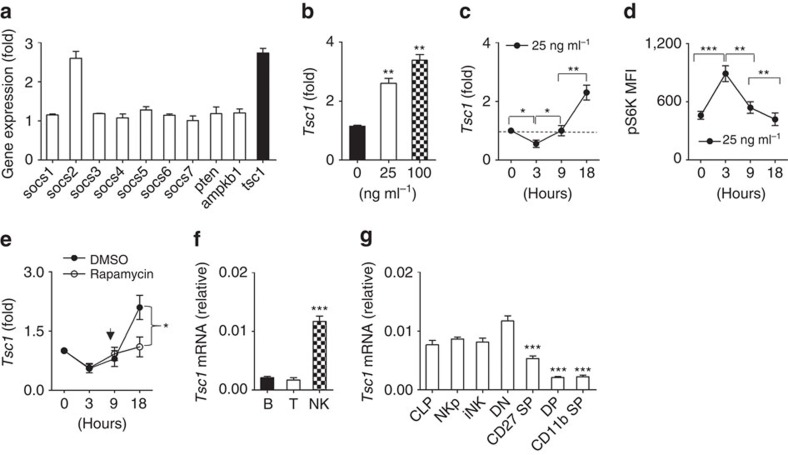
Dynamic *Tsc1* expression following IL-15 stimulation. (**a**–**c**) Quantitative reverse transcription-PCR (RT-PCR) analysis of the indicated genes in sorted CD3^−^NK1.1^+^ cells from the spleen of WT mice before and after stimulation with 25 ng ml^−1^ IL-15 for 18 h (**a**), various concentration of IL-15 (**b**), or at the indicated time points (**c**). (**d**) Intracellular phosphorylated S6 in sorted NK cells after stimulation with 25 ng ml^−1^ IL-15 was detected by flow cytometry at the indicated time points, and the mean fluorescence intensity was calculated. (**e**) *Tsc1* messenger RNA (mRNA) expression was analysed by quantitative RT-PCR in sorted CD3^−^NK1.1^+^ cells after stimulation with 25 ng ml^−1^ IL-15 for 18 h in the presence of DMSO or rapamycin (10 nM). (**f**) Analysis of *Tsc1* mRNA expression in sorted T, B and NK cells by quantitative PCR. **(g)** Analysis of *Tsc1* mRNA expression in CLP, NKp and immature NK cells (iNK), and NK cell subsets, including CD27^−^CD11b^−^(DN), CD27^+^CD11b^−^(CD27 SP), CD27^+^CD11b^+^ (DP) and CD27^−^CD11b^+^ (CD11b SP), by quantitative PCR. The results were normalized to β-*actin* (**f**,**g**) or are presented relative to expression in untreated cells, which was set as 1 (**a**–**c**,**e**). Value represent mean±s.d. *P<0.05, **P<0.01 and ***P<0.001. Unpaired Student's *t*-tests (two-tailed) was used to calculate these values. All data represent at least three independent experiments.

To investigate how IL-15-mediated signalling is negatively controlled, we measured the messenger RNA levels of several potential suppressors involved in the JAK-STAT pathway and PI3K/mTOR pathway in NK cells stimulated with a high dose of IL-15 for 18 h (h). As expected, *Socs2*, a negative regulator of the JAK-STAT pathway, was found to be upregulated, as previously reported^9^. Notably, the expression of *Tsc1* was increased by over twofold after IL-15 triggering (Fig. 1a). The stimulation of NK cells by a gradient concentration of IL-15 resulted in a dose-dependent increase in *Tsc1* expression (Fig. 1b). An in-depth analysis demonstrated that *Tsc1* expression was slightly suppressed 3 h after IL-15 stimulation but then gradually increased at the later time points (9–18 h; Fig. 1c). To examine whether the IL-15-induced changes in *Tsc1* expression correlates with mTOR activity, the phosphorylation of S6K (pS6K), an indicator of mTORC1 activation, was measured. *Tsc1* expression was decreased at the earliest time of IL-15 stimulation (3 h) whereas pS6K was upregulated. At 9–18 h after IL-15 stimulation, however, *Tsc1* expression was increased whereas pS6K was downregulated to the baseline level (Fig. 1d). Taken together, these results indicate that Tsc1 likely acts as a negative regulator to prevent prolonged IL-15-induced mTORC1 activation.

To understand how the dynamic regulation of *Tsc1* in response to IL-15 was achieved, *Tsc1* expression was monitored after treatment with rapamycin, an inhibitor of mTORC1. This treatment significantly counteracted the upregulation of *Tsc1* by IL-15 (Fig. 1e). Therefore, the increased expression of *Tsc1* is dependent on mTORC1 activation.

### *Tsc1* is mainly expressed in immature NK cells

To understand the physiological role of Tsc1 in NK cell development, the expression levels of *Tsc1* were compared among the three major types of lymphocytes. Compared with T and B lymphocytes, *Tsc1* was highly expressed in common lymphoid progenitor (CLP) and NK cells (Fig. 1f,g), mainly NKp, as well as relatively immature NK cells (CD27^*−*^CD11b^*−*^). The *Tsc1* level was found to gradually decrease with NK cell maturation (Fig. 1g). The dynamic expression of *Tsc1* suggests that this protein might be involved in IL-15-regulated early NK cell differentiation.

### Tsc1 deletion affects the number of T and B cells

We first generated haematopoietic-specific *Tsc1*-deleted mice, *Tsc1*^*fl/fl*^*/Vav1-Cre*^*+*^ (referred to as *Tsc1*^−/−^ mice). The efficiency of *Tsc1* deletion was confirmed by quantitative PCR (Supplementary Fig. 1a). Given that two previous studies have established that inducible knockdown of *Tsc1* can lead to abnormal hematopoietic stem cell (HSC) numbers^25^,^26^, we initially determined whether *Tsc1* deletion affected the generation of HSCs and CLPs in our model. The total numbers of *Tsc1*-deleted bone marrow cells was much lower than in wild-type (WT) mice (Supplementary Fig. 1b); the percentage of HSCs in *Tsc1*-deleted mice were higher, but the absolute numbers of HSCs were slightly lower than in WT mice. Despite this observation, the absolute numbers of CLPs were comparable between the two genotypes (Supplementary Fig. 1c–e), which allowed us to study lymphocyte development in the *Tsc1*^−/−^ mice.

The numbers of T and B cells were first measured. The *Tsc1*^−/−^ mice had considerably fewer T cells (including CD1d-tetramer-reactive NKT cells) in the spleen (Supplementary Fig. 2a,b), and the residual *Tsc1*-deficient T cells had an expanded CD62L^lo^CD44^hi^ population with an activated or memory phenotype (Supplementary Fig. 1c); however, the CD4^+^ and CD8^+^ T-cell subsets displayed normal selection in the *Tsc1*^−/−^ thymus and had comparable numbers of maturation-related markers, such as CD62L and CD69 (Supplementary Fig. 1d,e), suggesting that Tsc1 deficiency affects T-cell homeostasis but not development, as previously reported^21^. We further revealed that the *Tsc1*^−/−^ mice had normal number of pro-B cells, but they had reduced numbers of pre-B, immature and mature B cells, suggesting that the deletion of *Tsc1* blocked B-cell development at an early stage (Supplementary Fig. 3). Taken together, Tsc1 has different roles in the development and homeostasis of adaptive immune cells.

### Tsc1-deficient mice have a minimal pool of NK cells

**Figure 2:**
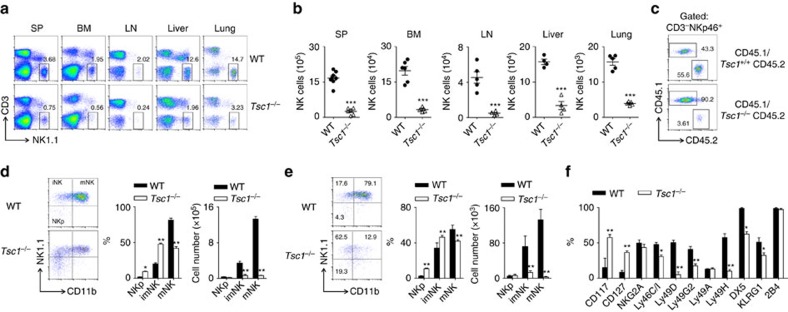
Tsc1 deficiency affects immature NK cell development. (**a**) Representative flow cytometric profiles of NK cells (CD3^−^NK1.1^+^) in the spleens (SP), bone marrow (BM), lymph nodes (LNs), livers, and lungs of WT and *Tsc1*^−/−^ mice. (**b**) The absolute number of NK cells in the indicated tissues and organs from the WT and *Tsc1*^−/−^ mice. Each symbol represents an individual mouse. (**c**) Lin^−^CD122^+^NK1.1^−^ NKp cells (2 × 10^5^) from WT or *Tsc1*^−/−^ CD45.2 mice were mixed with cells from WT CD45.1 mice at a 1:1 ratio and were transferred into sub-lethally irradiated *Rag1*^−/−^γ*c*^−^ recipient mice. CD45.1 versus CD45.2 expressions on gated splenic CD3^−^NKp46^+^ cells were detected by flow cytometry 8 weeks after the BM reconstitution. (**d**,**e**) Left, representative flow cytometry plots (Left) show the percentages of NKp (NK1.1^−^CD11b^−^), imNK (NK1.1^+^CD11b^−^) and mNK (NK1.1^−^CD11b^+^) cells on gated CD3^−^CD122^+^ splenocytes (**d**) and bone marrow cells (**e**) from WT and *Tsc1*^−/−^ mice. The numbers are percentages of the indicated quadrants among the gated CD3^−^CD122^+^ cells. Right, the absolute number of NKp, imNK and mNK cells in splenic (**d**) and bone marrow (**e**) cells were quantified. (**f**) Flow cytometry analysis of development-related NK cell receptors on NK1.1^+^B220^−^CD3^−^ cells in the spleen. The numbers indicate percentages of receptor-positive NK cells. All data represent at least three independent experiments and calculated data are shown as means±s.d. *P<0.05, **P<0.01 and ***P<0.001. Unpaired Student's *t*-tests (two-tailed) was used to calculate these values.

Next, we focused on the roles of Tsc1 in innate NK cell physiology. Notably, the NK cell numbers were reduced by over 90% in the spleens and bone marrow of the *Tsc1*^−/−^ mice (Fig. 2a,b). Remarkable reductions in the numbers of NK cells were also observed in other organs, including the lymph nodes, liver and lungs (Fig. 2a,b). To address whether the diminished NK cell pools in the *Tsc1*^−/−^ mice were cell-autonomous defects, and were not due to the possible defect of HSC generation, Lin^−^CD122^+^NK1.1^−^ NKp cells isolated from CD45.1^+^ WT and CD45.2^+^ *Tsc1*^−/−^ mice were mixed at a 1:1 ratio, then adoptively transferred into immunodeficient *Rag1*^−/−^γ*c*^−^ mice. NKp cells from the CD45.1^+^ mice showed much greater repopulation activity than those from the *Tsc1*^−/−^ mice (Fig. 2c). Therefore, the critical requirement of Tsc1 for NK cell development is cell intrinsic.

### Tsc1 regulates immature NK cell development

NK cells sequentially acquire differentiation markers with their maturation. Based on these markers, NK cell development is divided into multiple stages. *Tsc1*^−/−^ mice had comparable numbers of NKp cells. The proportion of early CD3^−^CD122^+^NK1.1^+^CD11b^−^ immature NK cells was much higher in the *Tsc1*^−/−^ mice, but their absolute numbers were dramatically reduced (Fig. 2d,e). The proportions and absolute numbers of mature NK cells that are CD3^−^CD122^+^NK1.1^+^CD11b^+^ were significantly decreased in the *Tsc1*^−/−^ mice (Fig. 2d,e). Analysis of NK cell receptors demonstrated that the proportion of NK cells expressing Ly49 family members (including Ly49C, D, G and H) and integrin DX5 was minimal in the *Tsc1*^−/−^ mice, whereas the proportion of NK cells with immature markers (either CD117 or CD127) was markedly higher (Fig. 2f). These data collectively suggest that NK cell development in the *Tsc1*^−/−^ mice was blocked at an early stage.

### Tsc1-deficient mice have defective innate immunity

**Figure 3:**
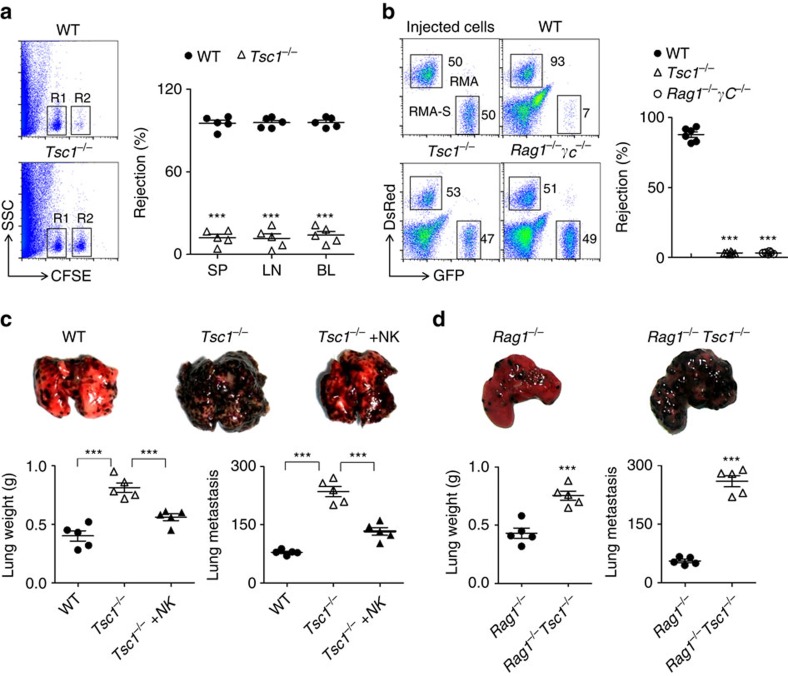
Tsc1 deficiency impairs NK cell-mediated immunosurveillance. (**a**) Left, representative flow cytometry plot of carboxyfluorescein diacetate succinimidyl ester (CFSE)-positve cells obtained from the spleens of the indicated recipient mice at 18 h after injection with an equal number of WT or *β2m*-deficient splenocytes labelled with various concentrations of the cytosolic dye CFSE. R1, CFSE-low splenocytes from WT mice; and R2, CFSE-high splenocytes from *β2 m*-deficient mice. Right, the percentages of rejected *β2m*-deficient splenocytes from the spleens (SP), lymph nodes (LN) and blood (BL) of the indicated recipient mice. (**b**) Left, representative flow cytometry plot of injected RMA-S cells in the peritoneal cavity at 18 h after intraperitoneal injection of the indicated mice with a mixture of NK cell-sensitive RMA-S cells expressing green fluorescent protein (GFP) together with NK cell-resistant RMA cells expressing the fluorescent protein DsRed. Immunocompromised *Rag1*^−/−^γ*c*^−^ mice were employed as controls. The injected cells are shown. Right, the percentage of rejected RMA-S cells. (**c**,**d**) B16 metastasis assay, the indicated mice were injected intravenously with 2 × 10^5^ B16 cells. The mice were killed 14 day later, and the lung weights and numbers of tumour nodules were counted. To prove NK-cell dependence, 2** × **10^6^ of purified NK cells from WT mice were adoptively transferred to *Tsc1*^−/−^mice (‘*Tsc1*^−/−^+NK' in **c**). Each symbol represents an individual mouse. Data are shown as means±s.d. ***P<0.001. Unpaired Student's *t*-tests (two-tailed) was used to calculate these values.

To confirm the diminished NK-mediated functions in the *Tsc1*^−/−^ mice, the ability of NK cells to mediate ‘missing-self' rejection was evaluated, as previously described^27^,^28^. We found that MHC-I-missing splenocytes were largely eliminated by NK cells in control mice; however, NK cell-mediated killing was barely detectable in *Tsc1*^−/−^ mice (Fig. 3a). We further examined the NK cell-mediated *in vivo* rejection of RMA-S cells, an NK-sensitive tumour cell line, and observed that the severe defect in the *Tsc1*^−/−^ mice was nearly comparable to that in immunocompromised mice (Fig. 3b). Lastly, we analysed whether *Tsc1* deletion affected the ability of NK cells to prevent the metastasis of B16 melanoma. Notably, the lung metastasis of B16 cells in *Tsc1*^−/−^ mice was much severer than that in control mice, as evidenced by the increases in the lung weights and numbers of tumour colonies in the *Tsc1*^−/−^ mice (Fig. 3c). When WT NK cells were adoptively transferred into the *Tsc1*^−/−^ mice, the defective phenotype was largely rescued, further confirming that *Tsc1*^−/−^ mice have severe defects in NK cell function (Fig. 3c). To exclude the possibility that the NK cell defect was extrinsically caused by altered T-cell homeostasis (Supplementary Fig. 2), *Tsc1*^−/−^ mice were further backcrossed onto *Rag1*-deficient mice. *Tsc1* deficiency in these mice led to severe defect in the anti-metastasis of B16 melanoma, even in the absence of T and B cells (Fig. 3d). Thus, *Tsc1*^−/−^ mice effectively lack peripheral NK cells and innate cytotoxic activity *in vivo*.

### Tsc1 restricts the proliferation of immature NK cells

**Figure 4:**
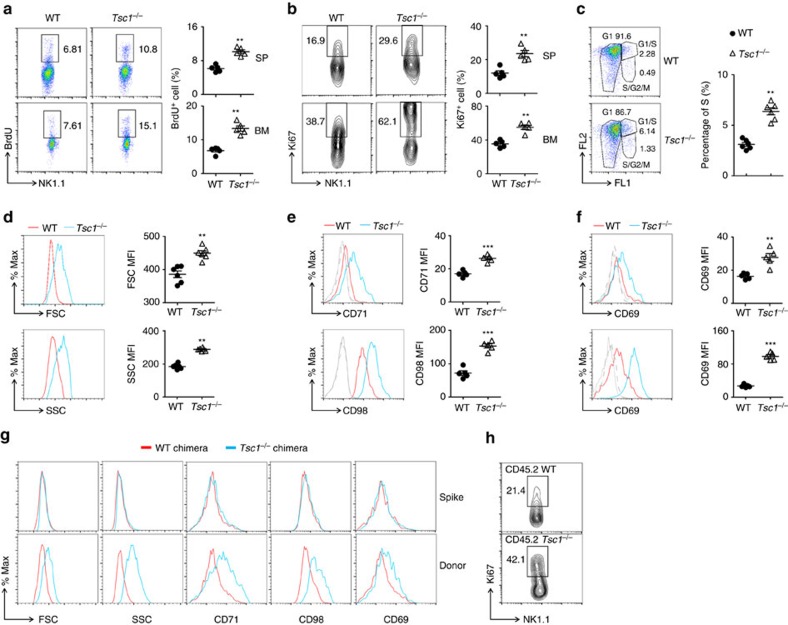
Tsc1-deficient NK cells exhibit increased proliferation and mTORC1 activity (**a**,**b**) Expression of the proliferation markers BrdU (**a**) and Ki67 (**b**) in SP and bone marrow (BM) NK cells from WT and *Tsc1*^−/−^ mice was assessed by intracellular flow cytometry (left), and the frequencies of BrdU^+^ or Ki67^+^ cells among the NK cells were calculated (right). (**c**) Representative flow cytometric plots (left) and the percentage of NK cells (right) in the S, G2 and M phases of the cell cycle, as determined using Fucci-492 mice, in which cells in the S, G2 and M phases of the cell cycle were fluorescent. The numbers near the indicated square boxes show the respective percentages of CD3^−^NK1.1^+^ cells. (**d**) The size (FSC) and cell granularity (SSC) of freshly isolated NK cells from WT and *Tsc1*^−/−^ mice were determined by flow cytometry (left), and the mean fluorescence intensity (MFI) was calculated (right). (**e**,**f**) Expression of the activation markers CD71, CD98 (**e**) and CD69 (**f**, upper, spleen; lower, BM) was assessed by flow cytometry, and the MFI was calculated. Each symbol represents an individual mouse. Data are shown as means±s.d. (**g**,**h**) BM cells from WT or *Tsc1*^−/−^ CD45.2 mice were mixed with cells from CD45.1 mice at a 1:1 ratio and were transferred into irradiated *Rag1*^−/−^γc^−^ recipients. The sizes (FSC), granularity (SSC) and the expression of CD71, CD98, CD69 (**g**) and the proliferation marker Ki67 (h) of splenic NK cells from spike BM derived (CD45.1^+^) and WT or *Tsc1*^−/−^ donor BM-derived (CD45.2^+^) cells in the reconstituted mixed chimeras were analysed. **P<0.01 and ***P<0.001. Unpaired Student's *t*-tests (two-tailed) was used to calculate these values.

To further determine whether the low number of NK cells in the *Tsc1*^−/−^ mice resulted from the reduced capacity for cell proliferation, we assessed the *in vivo* cycling of NK cells. Unexpectedly, *Tsc1* deletion resulted in a twofold increase in the frequency of proliferating cells, as determined by the incorporation of bromodeoxyuridine (BrdU) and by staining with the proliferation marker Ki67 under steady-state conditions (Fig. 4a,b). Using Fucci-2 reporter mice, in which cells in the S, G2 and M phases of the cell cycle were fluorescent^29^, we observed that 5–9% of *Tsc1*^−/−^ NK cells exhibited proliferating activity compared with 3% of WT NK cells (Fig. 4c). Therefore, Tsc1 is a negative regulator of NK cell proliferation.

In agreement with this finding that *Tsc1*^−/−^ NK cells proliferate rapidly, freshly isolated *Tsc1*^−/−^ NK cells were consistently larger size and showed more cells containing (Fig. 4d). Furthermore, the expression levels of the nutritional markers CD71 and CD98, as well as that of the activation marker CD69, were upregulated in these cells (Fig. 4e,f). Further experiments using mixed bone marrow chimera assays showed that the constitutive activation in the *Tsc1*^−/−^ mice was NK cell intrinsic (Fig. 4g,h). The phenotype was also observed in *Tsc1*^−/−^*Rag1*^−/−^ mice (Supplementary Fig. 4a,b). Thus, Tsc1 regulates NK cell development, likely by restricting the proliferation of immature NK cells.

### Constitutive mTORC1 activation disrupts NK cell development

**Figure 5:**
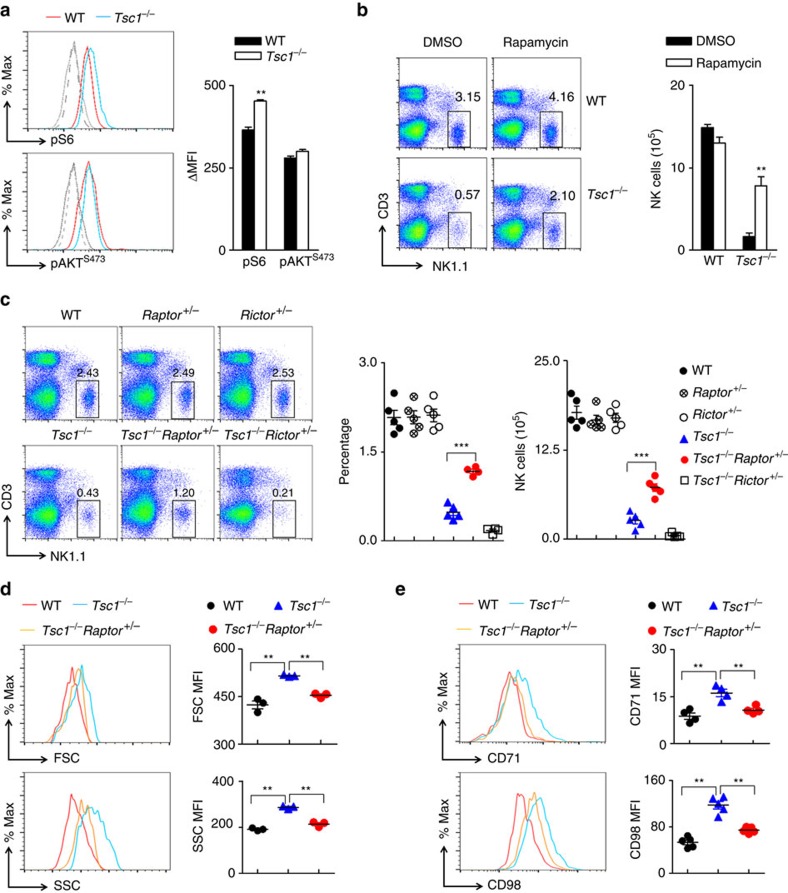
Constitutive activation of mTORC1 is detrimental to NK cell development. (**a**) Intracellular phosphorylated S6 and AKT^S473^ in splenic NK cells from WT and *Tsc1*^−/−^ mice were detected by flow cytometry. Representative overlaid histogram are shown (left panels). WT (grey dash lines) or *Tsc1*^−/−^(grey solid lines) NK cells stained with isotype antibody; WT (red lines) or *Tsc1*^−/−^ (blue lines) NK cells stained with the antibodies against pS6 (up) or AKT^S473^ (bottom). The mean fluorescence intensity (MFI) was calculated (right panels). (**b**) Left, representative flow cytometric plots showing the percentages of CD3^−^NK1.1^+^ NK cells in spleens from WT and *Tsc1*^−/−^ mice administered a mock injection (DMSO) or treated with a daily injection of rapamycin for 5 days. The numbers near the indicated square boxes show the percentages of CD3^−^NK1.1^+^ cells. Right panel, the absolute numbers of CD3^−^NK1.1^+^ NK cells in the spleens of the indicated mice. (**c**,**d**) Representative flow cytometric plots showing CD3^−^NK1.1^+^ NK cells in the spleens of the indicated mice. The quantification of percentages and absolute numbers of CD3^−^ NK1.1^+^ (right panel) is also presented. (**d**,**e**) FSC and SSC (**d**), CD71 and CD98 (**e**) on freshly isolated NK cells from the indicated mice were detected by flow cytometry, and the MFI was calculated. Each symbol represents an individual mouse. Data are showed as mean±s.d. and are representative of two independent experiments. **P<0.01 and ***P<0.001. Unpaired Student's *t*-tests (two-tailed) was used to calculate these values.

We next determined the biochemical mechanisms by which Tsc1 controls NK cell quiescence. We first measured the effect of *Tsc1* deficiency on mTORC1 activity by examining phosphorylation of the ribosomal protein S6. Freshly isolated *Tsc1*^−/−^ NK cells exhibited an increase in the basal activity of this conventional mTORC1 target compared with naïve WT NK cells. However, *Tsc1*^−/−^ NK cells had the same level of phosphorylation of Akt at Ser473, which is phosphorylated by mTORC2 (Fig. 5a). To assess whether the increased activity of mTORC1 disrupted the dormancy programme in *Tsc1*^−/−^ NK cells, *Tsc1*^−/−^ mice were administered with rapamycin for 5 consecutive days. The chemical inhibition of mTORC1 resulted in the partial recovery of the percentage and absolute number of NK cells in the *Tsc1*^−/−^ mice (Fig. 5b), indicating that Tsc1 has an inhibitory effect on mTORC1 activity in NK cells.

To genetically confirm the requirement of optimal mTORC1 activity for early NK cell differentiation, we generated *Tsc1*^−/−^ mice that were heterozygous for *Raptor*, a scaffold protein of mTORC1 (*Tsc1*^*fl/fl*^*/Raptor*^*fl/+*^*/Vav1-Cre*^*+*^ mice, referred to as *Tsc1*^−/−^*Raptor*^*+/−*^ mice in brief), or for Rictor, which is a major component of mTORC2 (*Tsc1*^*fl/fl*^*/Rictor*^*fl/+*^*/Vav1-Cre*^*+*^ mice, referred to as *Tsc1*^−/−^*Raptor*^*+/−*^ mice in brief). The genetic knockdown of *Raptor* markedly increased the percentage and absolute number of NK cells in the *Tsc1*^−/−^ mice. However, the inactivation of a single allele of *Rictor* exacerbated the defective NK cell development in *Tsc1*^−/−^ mice, suggesting that mTORC2 plays a role in NK cell development (Fig. 5c). Furthermore, the partial inactivation of mTORC1 activity appeared to attenuate the increased activation in *Tsc1*^−/−^ NK cells (Fig. 5d,e). These results clearly demonstrate that the exacerbated mTORC1 activity in *Tsc1*^−/−^ mice is detrimental to the early development of immature NK cells.

### Tsc1 deficiency decreases the expression of Eomes and CD122

**Figure 6:**
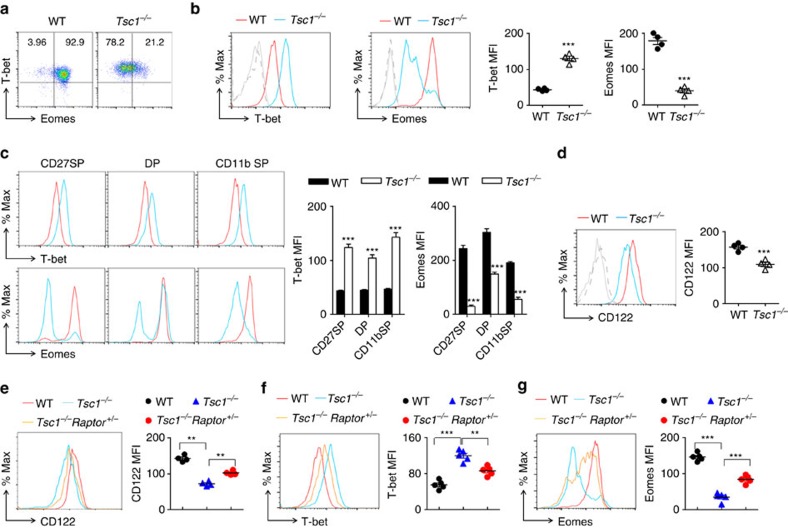
Aberrant expression of T-bet, Eomes and CD122 in *Tsc1*-deficient NK cells. (**a**) Representative flow cytometry plot showing intracellular staining of T-bet and Eomes in splenic NK cells from WT and *Tsc1*^−/−^ mice. (**b**,**c**) Representative overlaid histograms showing T-bet and Eomes expression by the indicated NK cells (**b**, left) or NK cell subsets (**c**, left), grey solid lines, WT isotype; grey dash lines, *Tsc1*^−/−^ isotype; red lines, WT NK cells stained with T-bet or Eomes antibody; and blue lines, *Tsc1*^−/−^ NK cells stained with T-bet or Eomes antibody. Quantifications of mean fluorescence intensity (MFI) are shown (right panel). (**d**,**e**) Representative overlaid histograms showing CD122 expression on the indicated NK cells. Grey solid lines, WT isotype; grey dash lines, *Tsc1*^−/−^ isotype; WT (red lines) or *Tsc1*^−/−^ (blue lines○NK stained with CD122 antibody. and quantification of MFI is shown (right panel). (**f**,**g**) Measurement of T-bet and Eomes by intercellular staining. Similar to **b**. Data represent the mean±s.d. of four mice are representative of three independent experiments. **P<0.01 and ***P<0.001. Unpaired Student's *t*-tests (two-tailed) was used to calculate these values.

Metabolic activation plays an essential role in T-cell differentiation by regulating the expression of transcription factors^30^. Thus, we sought to determine whether the over-activation of mTORC1 altered the expression profile of transcription factors in *Tsc1*^−/−^ mice. Intracellular staining of T-bet and Eomes, two T-box family transcription factors involved in the NK cell development, was performed (Fig. 6a). T-bet was over-expressed in *Tsc1*^−/−^ NK cells compared with Tsc1-intact NK cells. By contrast, the amount of Eomes was dramatically reduced in Tsc1-null NK cells, which was more apparent in relatively immature CD27^+^CD11b^−^ NK cells (Fig. 6b,c). This finding seems to be consistent with a previous study showing that T-bet and Eomes could antagonize one another's expression in NK cells^31^. To avoid confounding effects of comparing NK cell development in two different genotypes, we also examined the expression of the two transcription factors in a developmentally matched comparison, which revealed that *Tsc1*^−/−^ NK cells exhibited more T-bet, but less Eomes, at all stages (Fig. 6c). Thus, *Tsc1*-deficient NK cells had a deficiency in the expression of transcription factors involved in NK cell development.

Eomes regulates NK cell developments mainly through transcriptional regulation of CD122, such that *Eomes*-deficient NK cells have less CD122 (ref. 32). These previous data motivated us to examine CD122 level in *Tsc1*^−/−^ NK cells. As expected, CD122 expression was dramatically reduced in *Tsc1*^−/−^ NK cells (Fig. 6d). The decreased amount of CD122 in *Tsc1*^−/−^ NK cells could largely be rescued by the partial inactivation of mTORC1 in *Tsc1*^−/−^ *Raptor*^*+/−*^mice (Fig. 6e). Moreover, knocking-down *Raptor* expression was also able to significantly correct the aberrant expression of T-bet and Eomes (Fig. 6f,g). Therefore, over-activation of mTORC1 in *Tsc1*^−/−^ NK cells leads to an imbalance of T-bet and Eomes. In addition, the decreased Eomes expression most likely correlates with the defective expression of CD122, a subunit of the IL-15 receptor that determines the sensitivity of NK cells to IL-15.

### Tsc1-deficient NK cells undergo apoptosis

**Figure 7:**
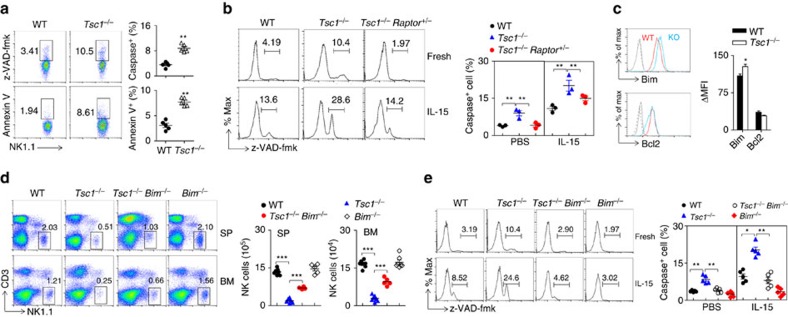
Tsc1-deficient NK cells undergo activation-induced apoptosis. (**a**) Left, representative flow cytometry plot showing Annexin V staining and caspase activity in NK cells from the indicated mice. The numbers adjacent to the outlined areas indicate the percentages of Annexin V- or caspase-positive cells. Right, quantification was performed. Each symbol represents an individual mouse; the small horizontal lines indicate the average (*n*=5 mice per group). The data are representative of two independent experiments. (**b**) Representative flow cytometry histograms showing caspase activity in freshly isolated or IL-15-stimulated NK cells from the indicated mice. The numbers adjacent to the outlined areas indicate the percentages of caspase-positive cells. Quantification was performed (right). (**c**) Intracellular staining of Bim and Bcl2 in naïve NK cells from the indicated mice (left). Grey solid lines, WT isotype; grey dash lines, *Tsc1*^−/−^ isotype; WT (red lines) or *Tsc1*^−/−^ (blue lines) NK stained with Bim or Bcl2 antibody. Quantifications of mean fluorescence intensity are shown in right panel. (**d**) Representative flow cytometric plots showing the percentages of NK cells in the spleens and bone marrow from the indicated mice (left). The numbers near the indicated square boxes show the respective percentages of CD3^−^NK1.1^+^ cells. Right, the absolute number of NK cells in the spleens and bone marrow from the indicated mice was calculated. (**e**) Representative histograms showing caspase activity in freshly isolated or IL-15-stimulated NK cells from the indicated mice (left). The numbers adjacent to the outlined areas indicate the percentages of caspase-positive cells. Quantification was performed (right). Each symbol represents an individual mouse. The data are representative of at least two independent experiments. Data are shown as mean±s.d. *P<0.05, **P<0.01 and ***P<0.001. Unpaired Student's *t*-tests (two-tailed) was used to calculate these values.

NK cells usually require high levels of CD122 to respond to IL-15. Deficient IL-15 signalling leads to Bim-dependent NK cell apoptosis^4^. We noted that *Tsc1*^−/−^ NK cells exhibited increased caspase activity and Annexin V staining, which are two hallmarks of apoptotic cell death (Fig. 7a). The increased apoptosis might result from the over-activation of mTORC1 because the reduction of *Raptor* could largely diminish the number of apoptotic NK cells in *Tsc1*^−/−^ mice (Fig. 7b). Interestingly, upon IL-15 stimulation, *Tsc1*^−/−^ NK cells were more likely to undergo apoptosis, whereas the apoptotic death of *Tsc1*^−/−^*Raptor*^*+/−*^ NK cells was much less pronounced (Fig. 7b). These data suggest that Tsc1 plays an important role in preventing activation-induced NK cell apoptosis.

The balance of pro- and anti-apoptotic members of the Bcl2 family controls the survival of immune cells. Among these members, Bim is considered one of important initiators of NK cell apoptosis^4^. Although the expression of anti-apoptotic Bcl2 was not significantly affected by *Tsc1* deficiency, *Tsc1*^−/−^ NK cells showed elevated Bim expression in the resting state (Fig. 7c). To determine whether *Tsc1*^−/−^ NK cells undergo Bim-dependent apoptosis, *Tsc1*^−/−^ mice were bred with *Bim*^*fl/fl*^ mice to yield *Tsc1*^*fl/fl*^*/Bim*^*fl/fl*^*/Vav1-Cre*^*+*^ mice (referred to as *Tsc1*^−/−^*Bim*^−/−^ mice). As a result, the additional deletion of Bim partially but significantly increased the frequency and absolute number of NK cells in *Tsc1*^−/−^*Bim*^−/−^ mice compared with those in *Tsc1*^−/−^ mice (Fig. 7d). Moreover, the removal of Bim largely counteracted the increased sensitivity of *Tsc1*^−/−^ NK cells to IL-15-induced apoptosis (Fig. 7e). However, the Bim deletion failed to correct the abnormal activation of *Tsc1*^−/−^ NK cells (Supplementary Fig. 5), suggesting that Bim-dependent NK cell death in *Tsc1*^−/−^ mice is most likely a consequence of the activation. Taken together, the Bim-dependent apoptotic pathway only partially mediates the death of *Tsc1*^−/−^ NK cells, indicating that other apoptosis pathways may exist^4^.

### Tsc1 is dispensable for NK cell terminal differentiation

**Figure 8:**
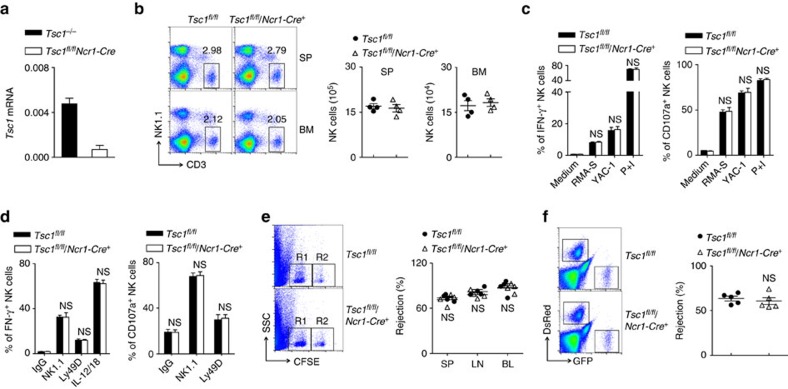
Tsc1 is not required for terminal NK cell differentiation or function. (**a**) Quantitative reverse transcription-PCR analysis of *Tsc1* expression in sorted CD3^−^NK1.1^+^ cells from indicated mice. Quantification was performed using data from two independent experiments (*n*=6). (**b**) Left panel, representative flow cytometric plots showing the percentages of CD3^−^NK1.1^+^ NK cells in the spleens (SP) and bone marrow from *Tsc1*^*fl/fl*^ and *Tsc1*^*fl/fl*^*Ncr1-Cre*^*+*^ mice. Right, absolute numbers of CD3^−^NK1.1^+^ NK cells in the spleens and bone marrow of the indicated mice. Each symbol represents an individual mouse. The data are representative of three independent experiments. (**c**) Splenic lymphocytes were prepared from poly I:C-treated mice and co-cultured with an equal number of tumour cells (RMA-S and YAC-1 cells); medium alone served as a negative control, and Phorbol-12-myristate-13-acetate (P) plus ionomycin (I) served as a positive control. Intracellular staining was performed to assess IFN-γ production (left). CD107a expression was chosen as a marker of NK cell degranulation (right). Data represent the mean±s.d. of 3–4 mice and are representative of two independent experiments. (**d**) Splenic lymphocytes were prepared from poly I:C-treated mice and stimulated with plate-coated antibodies or cytokine cocktail; The bar graphs show the average percentages of IFN-γ^+^ cells (left) and CD107a^+^ cells (right) for three experiments. Data represent the mean ±s.d. (**e**) NK-mediated rejection of MHC-I-deficient hematopoietic cells, as described in Fig. 3a. (**f**) NK-mediated rejection of RMA-S cells, as described in Fig. 3b. Each symbol represents an individual mouse; the small horizontal lines indicate the average (*n*=5 mice per group). The data are representative of three independent experiments. NS, non significant difference.

The above findings reveal a very unique role for Tsc1 in early NK cell differentiation. To further investigate whether Tsc1 is required for NK cell terminal differentiation and function, we generated a *Tsc1*^*fl/fl*^*/Ncr1-Cre*^*+*^mouse model in which Tsc1 was deleted during the late stage of NK cell development by the enzyme Cre, which is controlled by the promoter of *Ncr1*, which encodes NKp46 in mice. Cre expression in terminal NK cells lead to an 80% reduction of *Tsc1* level (Fig. 8a). The terminal deletion of Tsc1 did not result in a decrease in the percentage and number of NK cells (Fig. 8b). This model facilitated the assessment of the roles of Tsc1 in NK cell functions. Tsc1-deleted NK cells exhibited comparable levels of IFN-γ production and CD107a expression following stimulation with haematopoietic RMA-S and YAC-1 cell lines *in vitro* (Fig. 8c) and by plate-coated antibodies against the ITAM-containing receptor NK1.1 or Ly49D (Fig. 8d). These results were supported by the finding of Tsc1-null NK cell activation by the combination of the cytokines IL-12 and IL-18 (Fig. 8d). Moreover, an extensive *in vivo* analysis showed that the *Tsc1-*deleted mice successfully rejected the mismatched *β2m*^−/−^splenocytes and killed the RMA-S cells, comparable to the control mice (Fig. 8e,f).

To exclude the possibility that the dispensable activity of Tsc1 observed during the late stage of NK cell differentiation and function was due to the unsuccessful deletion of Tsc1 in the model, we further bred *Tsc1*^*fl/fl*^*Ncr1-Cre*^*+*^ mice with *e*YFP^STOP^ reporter mice to yield *Tsc1*^*fl/fl*^*/eYFP*^*STOP*^*/Ncr1-Cre*^*+*^ mice, in which the Cre-expressing NK cells were mostly eYFP-positive. *Tsc1* messenger RNA was hardly detectable in the eYFP^+^ *Tsc1*^*fl/fl*^ */Ncr1-Cre*^*+*^ NK cells (Supplementary Fig. 6a). Using this fate-mapping strategy, we failed to observe apparent differences in NK cell functions, such as cytokine production and degranulation, between the two genotypes when NK cells were triggered by several types of stimuli, including tumour cells, plate-coated antibodies and a cytokine cocktail (Supplementary Fig. 6). We therefore concluded that Tsc1 is not required for terminal NK cell development and effector functions.

## Discussion

NK cells are the most important innate lymphocytes that mediate tumour surveillance. A fundamental immunological question is how the development and homeostasis of NK cells are regulated at the metabolic and transcriptional levels^33^,^34^. Although IL-15 has been confirmed to be indispensable for these processes^35^,^36^ and multiple intracellular signalling pathways and transcription factors that promote NK cell development have been extensively studied, knowledge is limited regarding how these signalling networks are negatively regulated. In addition, NK cells are regulated to maintain their quiescence for immune homeostasis, but the manner by which their quiescence is carefully maintained also remains undefined. In this study, using two genetic mouse models, we dissected the critical role of Tsc1 in the maintenance of NK cell quiescence. The disruption of Tsc1 during the early stage of NK cell commitment (using Vav1-Cre) led to a large decrease in the number of NK cells. Intriguingly, inactivation of Tsc1 at the late stage (using Ncr1-Cre) had no obvious effect on terminal NK cell maturation or function. Therefore, our results reveal a unique role for Tsc1 in regulating NK cell development in the early stage by controlling IL-15-stimulated mTORC1 activation.

Human patients with Tsc1 deficiency have a higher tumour incidence; in fact, Tsc1 was originally regarded as a tumour repressor^37^,^38^, and it is intrinsically required for normal cells to suppress the undue activation of mTORC1, which is believed to have increased activity in most tumors^39^. The immune system plays an important role in the surveillance of malignancy. With increased understanding of the comprehensive roles of Tsc1 in immune cells, such as adaptive T and B cells, as well as in innate immune cells, including dendritic cells, it is reasonable to speculate that the increased tumour incidence in Tsc1-deficient patients is likely correlated with compromised antitumor immunity. Among the immune cells, NK cells are generally considered to be the innate lymphocytes responsible for the immunesurveillance of tumour cells. In this study, we discovered that the loss of Tsc1 caused a decrease in the number of NK cells and that this decrease was strongly associated with the lung metastasis of melanoma. Further research is required to determine whether NK cells are defective in patients with Tsc1 mutations, which may explain the high incidence of tumors in Tsc1-null patients. Therefore, Tsc1 may play an extrinsic role in the suppression of tumour growth via regulation of the cellularity of NK cells.

Emerging evidence obtained using mouse genetic models has revealed that IL-15-mediated metabolic activation is critical for NK cell development via the triggering of mTOR activation and the upregulation of nutritional receptors^13^,^18^,^40^. The NK cell-specific deletion of mTOR (Ncr1-cre generated by Eric Vivier's laboratory) markedly impaired NK cell differentiation and activation^18^. In addition, our recent results have revealed that the deletion of PDK1, an upstream mTOR kinase, causes severe defects in NK cell development^13^; however, the manner by which mTOR activation regulates NK cell development remains unknown. Further, it is unclear whether mTORC1 or 2 is involved in IL-15 signalling and how the distal signalling is negatively controlled. In the current study, Tsc1 expression was found to be dynamic following IL-15 stimulation. During the very early stage, IL-15 slightly inhibited Tsc1 expression, accompanied by an increase in mTORC1 activation. Thus, the transient downregulation of Tsc1 likely highlights a novel mechanism that facilitates transcriptional events promoting early NK cell development via a positive feedback loop involving PDK1-Mtor/E4BP4/Eomes/CD122 (ref. 13); however, continuous mTORC1 activation could increase Tsc1 expression during the late stage of IL-15 stimulation, thereby initiating a mechanism of feedback inhibition to suppress the prolonged mTOR activation. Therefore, we have identified a regulatory mechanism of IL-15 signalling that prevents excessive mTORC1 activation during NK cell development.

In T cells, the loss of Tsc1 could cause an increase in mTORC1 activation. Moreover, Tsc1-deficient T cells display decreased activation of mTORC2 (ref. 21). Despite this finding, emerging evidence suggests that increased mTORC1 activity but not decreased mTORC2 activity contributes to most of the observed phenotypes caused by Tsc1 deficiency^21^,^22^. Our data confirm that the biochemical inhibition of mTORC1 activity by rapamycin largely restores the defects observed during early NK cell differentiation in the absence of Tsc1, indicating that the control of mTORC1 activity by Tsc1 is indispensable for NK cell quiescence. Further evidence obtained from genetic experiments indicates that the removal of one allele of *Raptor* also largely diminishes the abnormal phenotype of Tsc1-deficient mice. Thus, the Tsc1-dependent control of mTORC1 is vital for the functioning of the innate and adaptive immune systems.

Although convincing evidence suggests that increased mTORC1 activity contributes to the disruption of early NK cell quiescence, we unexpectedly found that inactivation of a main component of mTORC2, *Rictor,* aggravated the developmental defects in Tsc1-deficient NK cells. This finding has two implications. First, it further confirms that Tsc1 regulates early NK cell differentiation independent of mTORC2 activation. Moreover, Rictor-containing mTORC2 may participate in early NK cell development in an mTORC1-independent manner. Thus, further studies involving the genetic inactivation of mTORC1 and mTORC2 will help to elucidate the multifaceted role of mTOR in NK cell development.

A previous study has reported that Tsc1 is critical for the regulation of T-cell quiescence and function^21^. Tsc1 deletion causes no obvious developmental defects in T cells, with the exception of NKT cells in the thymus; therefore, Tsc1 only affects the quiescence of terminally differentiated T cells. Similarly, Tsc1 is only involved in NKT cell terminal maturation through the regulation of T-box transcription factor expression^41^. Moreover, Tsc1 regulates effector functions; for example, Tsc1-deficient T cells also exhibit hyporesponsiveness to T-cell receptor triggering. In contrast, in this study, we have revealed a distinct role of Tsc1 in NK cell biology. The stage-specific deletion of Tsc1 allowed us to observe the dispensable role of Tsc1 in terminal NK cell differentiation and effector functions. NK cells with terminal deletion of Tsc1 exhibit normal survival and function, strongly suggesting that mTORC1 signalling is not strictly required for other receptors involving NK activation. Furthermore, although IL-15 is mandatory for NK cell survival and homeostasis^42^, terminal deletion of Tsc1 had no visible effect on peripheral NK cell homeostasis. In contrast, early deletion of Tsc1 caused severe defects in NK cell differentiation. These findings indicate that Tsc1 plays a unique role in IL-15-mediated mTORC1 activation, which is critical for NK cell development.

In summary, NK cell development in the early stage is tightly controlled by IL-15 signalling. Negative regulation of this process by Tsc1 is essential for NK cell development. Tsc1 deficiency causes the hyper-activation of mTORC1, which is detrimental to immature NK cell development. Thus, we have identified a unique checkpoint that is crucial for NK cell development. Given the importance of the adoptive transfer of NK cells in tumour immunotherapy, the optimal mTORC1 activation triggered by IL-15 may provide new strategies for the *ex vivo* expansion of NK cells.

## Methods

### Mice

The *Tsc1*^*flox/flox*^ mice were a gift from Dr. Hongbin Zhang (Beijing Union Medical College, China). *Tsc1*^*fl/fl*^ mice were backcrossed to C57BL/6 background for eight generations before being bred with the other mice. Haematopoietic- or NK cell-specific Tsc1-deficient mice were generated by crossing *Tsc1*^*fl/fl*^ with Vav1-Cre (B6.Cg-Tg(Vav1-cre)A2Kio/J, Jackson lab, stock Number, 008610) or Ncr1-Cre mice generated in our lab^13^. Fucci-2 reporter mice were a gift from Dr. Hai Qi (Tsinghua University) and crossed with *Tsc1*^*fl/fl*^*Vav1-Cre*^*+*^ mice to obtain Tsc1-deficient Fucci-2 transgenic mice, where cells in S, G2 and M phases of the cell cycle are fluorescent^29^. *Rag1*^−/−^γ*c*^*−*^mice was described previously^13^. *β2*m-deficient mice, C57BL/6 mice, CD45.1, *Rptor*^*flox/flox*^ mice, *Rictor*^*flox/flox*^ mice and *Bim*^*flox/flox*^ mice were purchased from Jackson lab. Both female and male mice with age of 8–12 weeks were used in our experiments. All the mice are C57BL/6 background and maintained under specific pathogen-free animal facilities of Tsinghua University. All procedures involving animals were approved by the Animal Ethics Committee of Tsinghua University.

### Flow cytometry

Flow cytometry was performed on a BD LSR II (four-laser Blue/Red/Violet/ultraviolet flow cytometry analyzer, BD Biosciences). Monoclonal antibodies against mouse CD3 (#48-0032, 17A2, 1:200), NKp46 ((#11-3351, 29A1.4, 1:100), NK1.1 (#17-5941, PK136, 1:200), CD117 (#48-1171, 2B8, 1:200), CD127 (#25-1273, SB/199, 1:200), Ly49A (#12-5856, A1, 1:200), Ly49C/I (#553277, 5E6, 1:200), Ly49H (#17-5886, 3D10, 1:200), Ly49G2 (#46-5781, 4D11, 1:200), CD135 (#12-1351, A2F10, 1:200), SCA-1 (#45-5981, D7, 1:200), NKG2D(#25-5882, CX5, 1:200), NKG2A/C/E(#46-5897, 16a11, 1:200), CD11b (#17-0112,M1/70, 1:200), CD27 (#12-0271, LG.7F9, 1:200), IFN-γ (#12-7311, XMG1.2, 1:200), CD107a (#50-1071, eBio1D4B, 1:100) and isotype controls were purchased from eBioscience (San Diego, CA) or BD Biosciences (Mississauga, Ontario, Canada). Anti-phospho-AKT (S473, #9271, 1:50), and Anti-phospho-S6 (#4856, 1:200) were obtained from Cell Signaling Technology (Beverly, MA). CD1d-PBS157 tetramer was kindly provided by NIH tetramer facility. For analysis of surface markers, cells were stained in PBS containing 2% (wt/vol) foetal bovine serum with indicated antibodies from eBioscience or BD. The expression level was presented as net mean fluorescence intensity (ΔMFI), which was determined by subtracting mean fluorescence intensity of isotype control. For detection of phosphorylated signalling proteins, NK cells were fixed with Phosflow Lyse/Fix buffer, followed by permeabilization with Phosflow Perm buffer III (BD) and staining with antibodies.

### Real-time PCR

Total RNA from splenocytes, bone marrow cells or FACS-sorted NK, T and B cell subsets was extracted using Trizol Kit (Invitrogen), and reverse-transcribed using reverse transcription system (Promega, A3500). Quantitative PCR was conducted using SYBR green-based detection. The expression level of the genes of interest was determined relative to the expression of β*-actin*.

### Detection of NK cell apoptosis

Splenocytes were stained with Annexin V (BD Biosciences), and Caspase activity was measured with fluorescein isothiocyanate-conjugated z-VAD-fmk according to the manufacturer's instruction (eBiosences).

### Adoptive cell transfer

A mixture of 2 × 10^5^ Lin^−^CD122^+^NK1.1^−^ NKp cells from WT or *Tsc1*^−/−^ CD45.2 mice were mixed with cells from CD45.1 mice at a 1:1 ratio and transferred into irradiated *RAG1*^−/−^γ*c*^−^ recipients. Reconstitution of recipients was assessed by flow cytometry at 8 weeks after transplantation.

### *In vitro* cell activation

Mice were treated with intraperitoneal injection of polyI:C (10 μg mg^−1^) for 18 h. Poly I:C-activated splenocytes (2 × 10^6^) were co-cultured with the same number of different target cells in a total volume of 500 μl in 24-well plate. For antibody stimulation, 24-well plates were coated with indicated antibodies at a concentration of 1 μg ml^−1^ overnight. For cytokine stimulation, Poly I:C-activated splenocytes were treated with cytokines (recombinant mouse IL-12 (10 ng ml^−1^), recombinant mouse IL-18 (10 ng ml^−1^)). BD GolgiStop reagent (BD Biosciences) was incorporated to inhibit intracellular protein transport processes, and meanwhile, eFluor-660-conjugated anti-CD107a antibody or respective control isotype was added at the beginning of incubation to detect lysosome synthesis. Splenocytes stimulated with PMA (50 ng ml^−1^) plus ionomycin (1 μM) stimulation were used as positive controls. Medium only was used as negative control. Four hours after stimulation, cell mixtures were harvested for detection of intracellular IFN-γ. First, the cells were stained for NKp46 (fluorescein isothiocyanate-NKp46) plus PE-Cy5-CD3, and then fixed/permeabilized with BD Cytofix/Cytoperm Buffer (BD Biosciences). These cells were then stained with anti-IFN-γ-PE, or PE-conjugated isotype control as negative control. Data were acquired using a BD LSR Fortessa flow cytometer, and analysed with Cell Quest software (BD Biosciences).

### *In vivo* splenocyte rejection assay

Splenocytes from *β2m*-deficient (*β2m*^−/−^) mice were depleted of red blood cells by Ficoll-Hypaque density gradient centrifugation, and then labelled with 5 μM CFSE (Molecular Probes). At the same time, splenocytes from WT mice were labelled with 0.5 μM CFSE (10-fold <*β2m*^−/−^ cells). Two types of CFSE-labelled splenocytes were mixed at 1:1 ratio. A mixture of 2 × 10^6^ splenocytes was intravenously injected into mice pre-treated with 200 μg Poly I:C for 18 h. 18 h later, CFSE-positive cells in blood, spleen and lymph nodes were determined by flow cytometry. To exclude the role of NK cells in the recipient mice, *RAG1*^−/−^γ*c*^−^ mice were used. The percentage of *β2 m*^−/−^ splenocyte rejection was calculated with the following formula: 100 × [1−(percentage of residual *β2m*^−/−^ population in total CFSE^+^ cells of experimental group/percentage of residual *β2m*^−/−^ population in total CFSE^+^ cells of *RAG1*^−/−^γ*c*^*−*^mice group)].

### *In vivo* RMA-S clearance assay

Mice treated with 200 μg Poly I: C for 18 h were intraperitoneally injected with a mixture of target cells, NK-sensitive RMA-S cells expressing green fluorescent protein (GFP; 10^6^) and NK-non-sensitive RMA expressing DsRed (10^6^). 18 h later the mice were killed, and cells in peritoneal cavity were collected by repeated washing with PBS containing 2 μM EDTA. After centrifugation, cells were resuspended in 1 ml PBS. The relative percentages of RMA-S and RMA cells were measured by flow cytometry. The percentage of RMA-S cell rejection was calculated with the following formula: 100 × [1−(percentage of residual GFP^+^ population in total GFP^+^ and DsRed^+^ of experimental group/percentage of residual GFP^+^ population in total GFP^+^ and DsRed^+^ cells of *RAG1*^−/−^γ*c*^*−*^mice group)].

### B16 melanoma lung metastasis mouse model

B16F10 melanoma cells in the log phase were resuspended in 1 × Hanks Balanced Salt Solution and intravenously injected into the mice (2 × 10^5^ cells per mouse). fourteen days later the mice were killed. The lung was weighted and the number of lung surface nodules was counted under a dissecting microscope.

### Statistical analyses

Unpaired Student's *t*-tests (two-tailed) were performed using the Prism software. A *P* value of <0.05 was considered significant. *P<0.05, **P<0.01 and ***P<0.001.

### Data availability

The data that support the findings of this study are available from the corresponding author upon request.

## Additional information

**How to cite this article**: Yang, M. *et al*. NK cell development requires Tsc1-dependent negative regulation of IL-15-triggered mTORC1 activation. *Nat. Commun.* 7:12730 doi: 10.1038/ncomms12730 (2016).

## Supplementary Material

Supplementary Information Supplementary Figures 1-6

## References

[b1] DiSanto J. P., Muller W., Guy-Grand D., Fischer A. & Rajewsky K. Lymphoid development in mice with a targeted deletion of the interleukin 2 receptor gamma chain. Proc. Natl Acad. Sci. USA 92, 377–381 (1995). 7831294 10.1073/pnas.92.2.377 PMC42743

[b2] Kennedy M. K. . Reversible defects in natural killer and memory CD8 T cell lineages in interleukin 15-deficient mice. J. Exp. Med. 191, 771–780 (2000). 10704459 10.1084/jem.191.5.771 PMC2195858

[b3] Deng Y. . Transcription factor Foxo1 is a negative regulator of natural killer cell maturation and function. Immunity 42, 457–470 (2015). 25769609 10.1016/j.immuni.2015.02.006 PMC4400836

[b4] Huntington N. D. . Interleukin 15-mediated survival of natural killer cells is determined by interactions among Bim, Noxa and Mcl-1. Nat. Immunol. 8, 856–863 (2007). 17618288 10.1038/ni1487 PMC2951739

[b5] Min-Oo G., Bezman N. A., Madera S., Sun J. C. & Lanier L. L. Proapoptotic Bim regulates antigen-specific NK cell contraction and the generation of the memory NK cell pool after cytomegalovirus infection. J. Exp. Med. 211, 1289–1296 (2014). 24958849 10.1084/jem.20132459 PMC4076589

[b6] Sathe P. . Innate immunodeficiency following genetic ablation of Mcl1 in natural killer cells. Nat. Commun. 5, 4539 (2014). 25119382 10.1038/ncomms5539

[b7] Imada K. . Stat5b is essential for natural killer cell-mediated proliferation and cytolytic activity. J. Exp. Med. 188, 2067–2074 (1998). 9841920 10.1084/jem.188.11.2067 PMC2212377

[b8] Eckelhart E. . A novel Ncr1-Cre mouse reveals the essential role of STAT5 for NK-cell survival and development. Blood 117, 1565–1573 (2011). 21127177 10.1182/blood-2010-06-291633

[b9] Lee S. H. . Suppressor of cytokine signaling 2 regulates IL-15-primed human NK cell function via control of phosphorylated Pyk2. J. Immunol. 185, 917–928 (2010). 20543098 10.4049/jimmunol.1000784

[b10] Nandagopal N., Ali A. K., Komal A. K. & Lee S. H. The critical role of IL-15-PI3K-mTOR pathway in natural killer cell effector functions. Front. Immunol. 5, 187 (2014). 24795729 10.3389/fimmu.2014.00187 PMC4005952

[b11] Tassi I. . p110gamma and p110delta phosphoinositide 3-kinase signaling pathways synergize to control development and functions of murine NK cells. Immunity 27, 214–227 (2007). 17723215 10.1016/j.immuni.2007.07.014

[b12] Guo H., Samarakoon A., Vanhaesebroeck B. & Malarkannan S. The p110 delta of PI3K plays a critical role in NK cell terminal maturation and cytokine/chemokine generation. J. Exp. Med. 205, 2419–2435 (2008). 18809712 10.1084/jem.20072327 PMC2556795

[b13] Yang M. . PDK1 orchestrates early NK cell development through induction of E4BP4 expression and maintenance of IL-15 responsiveness. J. Exp. Med. 212, 253–265 (2015). 25624444 10.1084/jem.20141703 PMC4322053

[b14] Tassi I. . Phospholipase C-gamma 2 is a critical signaling mediator for murine NK cell activating receptors. J. Immunol. 175, 749–754 (2005). 16002670 10.4049/jimmunol.175.2.749

[b15] Briercheck E. L. . PTEN is a negative regulator of NK cell cytolytic function. J. Immunol. 194, 1832–1840 (2015). 25595786 10.4049/jimmunol.1401224 PMC4319309

[b16] Leong J. W. . PTEN regulates natural killer cell trafficking *in vivo*. Proc. Natl Acad. Sci. USA 112, E700–E709 (2015). 25646418 10.1073/pnas.1413886112 PMC4343124

[b17] Gumbleton M., Vivier E. & Kerr W. G. SHIP1 intrinsically regulates nk cell signaling and education, resulting in tolerance of an MHC class I-mismatched bone marrow graft in mice. J. Immunol. 194, 2847–2854 (2015). 25687756 10.4049/jimmunol.1402930 PMC4355317

[b18] Marcais A. . The metabolic checkpoint kinase mTOR is essential for IL-15 signaling during the development and activation of NK cells. Nat. Immunol. 15, 749–757 (2014). 24973821 10.1038/ni.2936 PMC4110708

[b19] Park Y. . TSC1 regulates the balance between effector and regulatory T cells. J. Clin. Invest. 123, 5165–5178 (2013). 24270422 10.1172/JCI69751 PMC3859395

[b20] Xie D. L., Wu J., Lou Y. L. & Zhong X. P. Tumor suppressor TSC1 is critical for T-cell anergy. Proc. Natl Acad. Sci. USA 109, 14152–14157 (2012). 22891340 10.1073/pnas.1119744109 PMC3435231

[b21] Yang K., Neale G., Green D. R., He W. & Chi H. The tumor suppressor Tsc1 enforces quiescence of naive T cells to promote immune homeostasis and function. Nat. Immunol. 12, 888–897 (2011). 21765414 10.1038/ni.2068 PMC3158818

[b22] Wang Y. . Tuberous sclerosis 1 (Tsc1)-dependent metabolic checkpoint controls development of dendritic cells. Proc. Natl Acad. Sci. USA 110, E4894–E4903 (2013). 24282297 10.1073/pnas.1308905110 PMC3864282

[b23] Wu J. . Tuberous sclerosis 1 promotes invariant NKT cell anergy and inhibits invariant NKT cell-mediated antitumor immunity. J. Immunol. 192, 2643–2650 (2014). 24532578 10.4049/jimmunol.1302076 PMC3965184

[b24] Shrestha S. . Tsc1 promotes the differentiation of memory CD8+ T cells via orchestrating the transcriptional and metabolic programs. Proc. Natl Acad. Sci. USA 111, 14858–14863 (2014). 25271321 10.1073/pnas.1404264111 PMC4205612

[b25] Chen C. . TSC-mTOR maintains quiescence and function of hematopoietic stem cells by repressing mitochondrial biogenesis and reactive oxygen species. J. Exp. Med. 205, 2397–2408 (2008). 18809716 10.1084/jem.20081297 PMC2556783

[b26] Gan B. . mTORC1-dependent and -independent regulation of stem cell renewal, differentiation, and mobilization. Proc. Natl Acad. Sci. USA 105, 19384–19389 (2008). 19052232 10.1073/pnas.0810584105 PMC2593615

[b27] Dong Z. . The adaptor SAP controls NK cell activation by regulating the enzymes Vav-1 and SHIP-1 and by enhancing conjugates with target cells. Immunity 36, 974–985 (2012). 22683124 10.1016/j.immuni.2012.03.023

[b28] Dong Z. . Essential function for SAP family adaptors in the surveillance of hematopoietic cells by natural killer cells. Nat. Immunol. 10, 973–980 (2009). 19648922 10.1038/ni.1763

[b29] Sakaue-Sawano A. . Tracing the silhouette of individual cells in S/G2/M phases with fluorescence. Chem. Biol. 15, 1243–1248 (2008). 19101468 10.1016/j.chembiol.2008.10.015

[b30] Rao R. R., Li Q., Odunsi K. & Shrikant P. A. The mTOR kinase determines effector versus memory CD8+ T cell fate by regulating the expression of transcription factors T-bet and Eomesodermin. Immunity 32, 67–78 (2010). 20060330 10.1016/j.immuni.2009.10.010 PMC5836496

[b31] Gordon S. M. . The transcription factors T-bet and Eomes control key checkpoints of natural killer cell maturation. Immunity 36, 55–67 (2012). 22261438 10.1016/j.immuni.2011.11.016 PMC3381976

[b32] Intlekofer A. M. . Effector and memory CD8+ T cell fate coupled by T-bet and eomesodermin. Nat. Immunol. 6, 1236–1244 (2005). 16273099 10.1038/ni1268

[b33] Male V. & Brady H. J. Transcriptional control of NK cell differentiation and function. Curr. Top. Microbiol. Immunol. 381, 173–187 (2014). 24850220 10.1007/82_2014_376

[b34] Ikawa T. Genetic and epigenetic control of early lymphocyte development. Curr. Top. Microbiol. Immunol. 381, 1–20 (2014). 24850218 10.1007/82_2014_370

[b35] Ali A. K., Nandagopal N. & Lee S. H. IL-15-PI3K-AKT-mTOR: a critical pathway in the life journey of natural killer cells. Front Immunol. 6, 355 (2015). 26257729 10.3389/fimmu.2015.00355 PMC4507451

[b36] Finlay D. K. Metabolic regulation of natural killer cells. Biochem. Soc. Trans. 43, 758–762 (2015). 26551725 10.1042/BST20150116

[b37] Kajino K. & Hino O. TSC1 and TSC2 gene mutations in human kidney tumors. Contrib. Nephrol. 128, 45–50 (1999). 10597376 10.1159/000059974

[b38] Mak B. C. & Yeung R. S. The tuberous sclerosis complex genes in tumor development. Cancer Invest. 22, 588–603 (2004). 15565817 10.1081/cnv-200027144

[b39] Mieulet V. & Lamb R. F. Tuberous sclerosis complex: linking cancer to metabolism. Trends Mol. Med. 16, 329–335 (2010). 20605525 10.1016/j.molmed.2010.05.001

[b40] Marcais A. & Walzer T. mTOR: a gate to NK cell maturation and activation. Cell Cycle 13, 3315–3316 (2014). 25485573 10.4161/15384101.2014.972919 PMC4614870

[b41] Wu J. . iNKT cells require TSC1 for terminal maturation and effector lineage fate decisions. J. Clin. Invest. 124, 1685–1698 (2014). 24614103 10.1172/JCI69780 PMC3973110

[b42] Ranson T. . IL-15 is an essential mediator of peripheral NK-cell homeostasis. Blood 101, 4887–4893 (2003). 12586624 10.1182/blood-2002-11-3392

