# Supplementary material for: NK cell development requires Tsc1-dependent negative regulation of IL-15-triggered mTORC1 activation
Source: Nat Commun. 2016 Sep 7;7:12730. doi: 10.1038/ncomms12730 (PMC5023956; doi:10.1038/ncomms12730)
Supplement: Supplementary Information — Supplementary Figures 1-6 [file ncomms12730-s1.pdf]

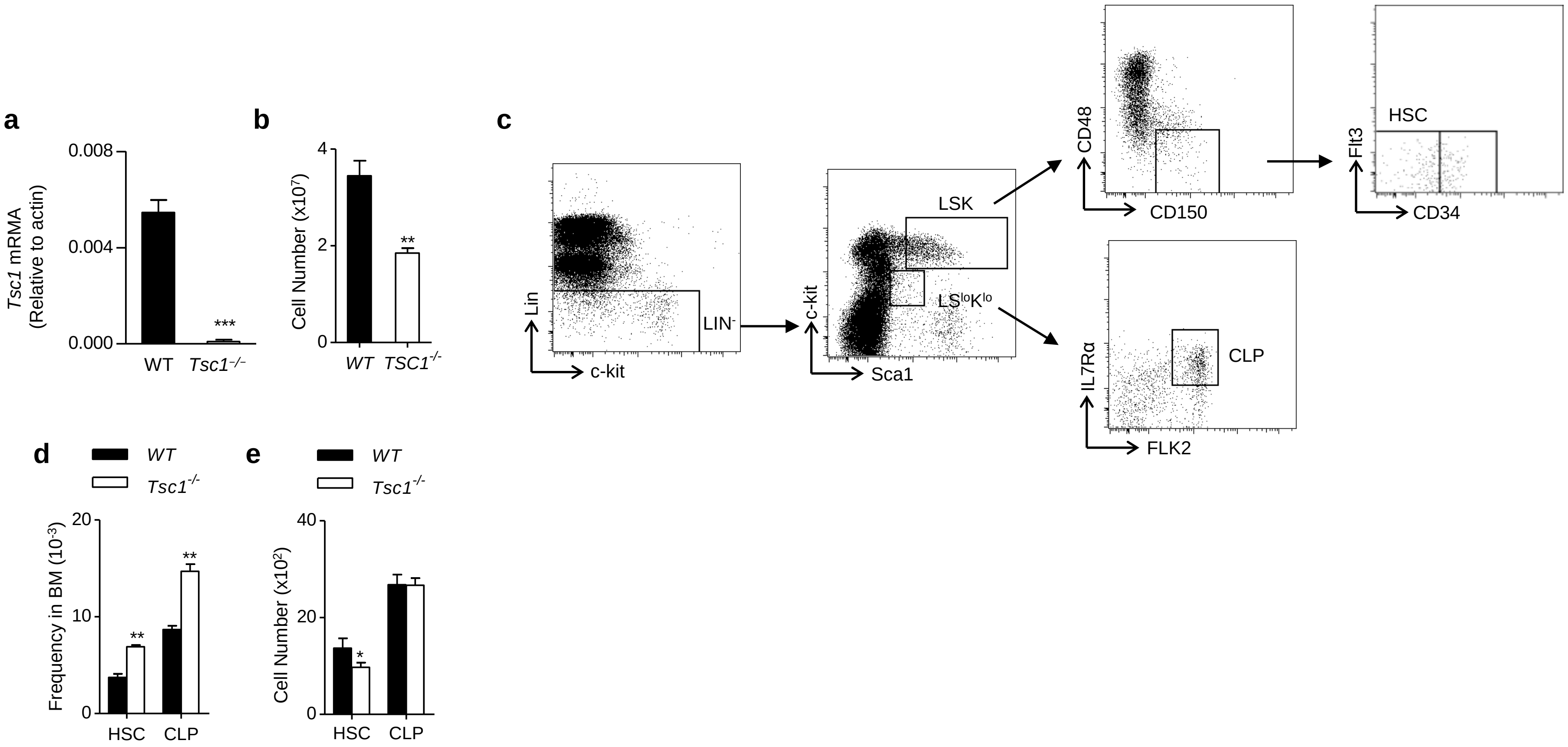

Supplementary Figure 1. Hematopoietic deletion of Tsc1 gave rise to less HSCs, but not CLPs.
(a). Total RNA was isolated from bone marrow cells of wild-type (WT) and *Tsc1*$^{-/-}$ mice. Tsc1 mRNA was analyzed by quantitative PCR. Data are shown as ratio of *Tsc1* to *β-actin*. (b). Absolute numbers of total BM cells in WT and *Tsc1*$^{-/-}$ mice. (c) Gating strategy for flow cytometry analysis of HSC and CLPs. (d,e). Frequencies and absolute numbers of HSC and CLPs in WT and *Tsc1*$^{-/-}$ mice. Values represent mean ± s.d and represent at least three independent experiments. **p* < 0.05, ** *p* < 0.01, ****p* < 0.001.

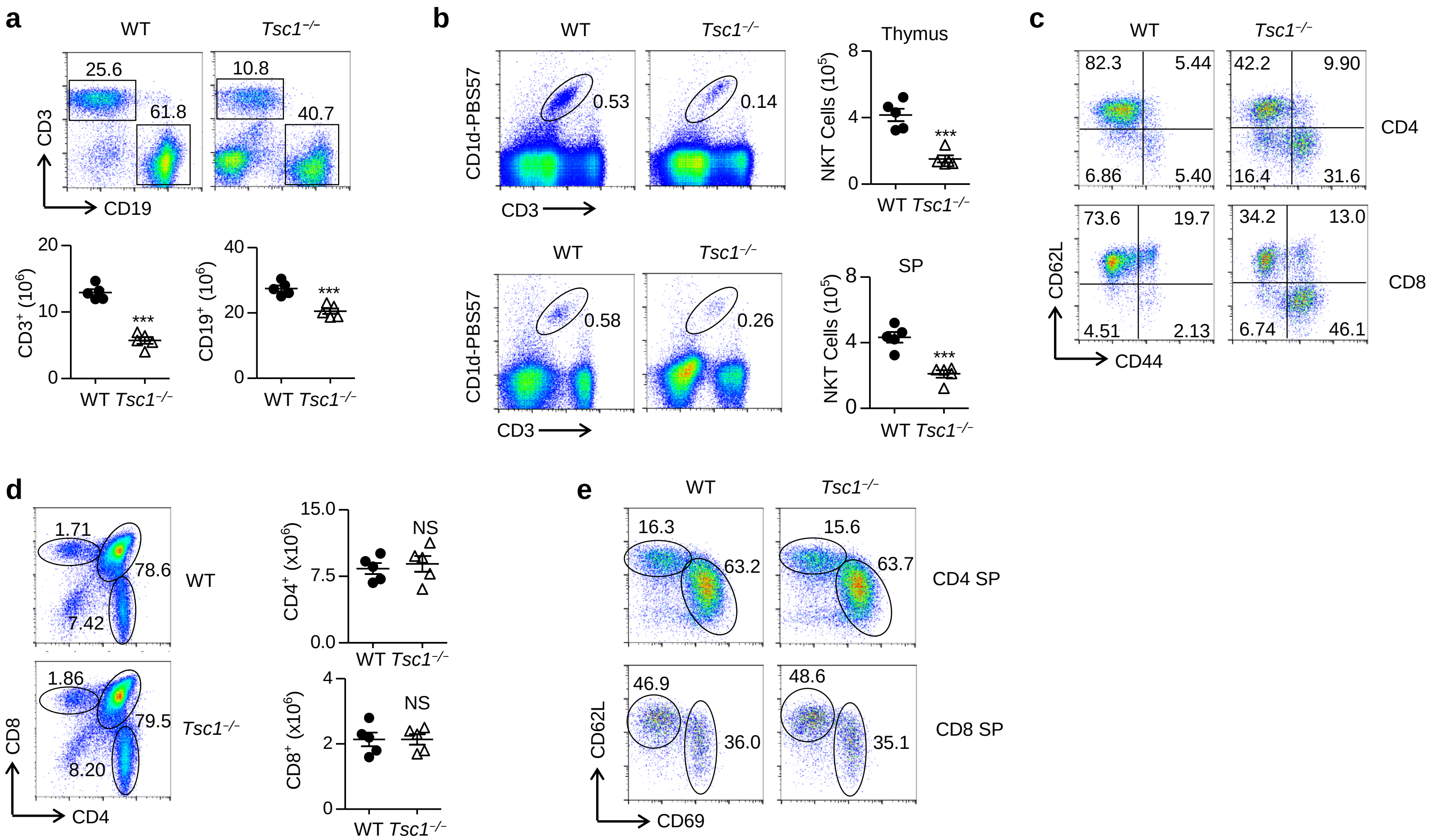

Supplementary Figure 2. Hematopoietic-specific deletion of Tsc1 disrupted peripheral T-cell hemostasis, rather than development.

(a) Representative flow cytometric plots showing CD3 vs CD19 expression on the splenocytes in WT and *Tsc1*$^{-/-}$ mice. Bottom, the absolute number of CD3^{+} T and CD19^{+} B cells in the spleens of wild-type and *Tsc1*$^{-/-}$ mice. (b) Analysis of iNKT cells (CD3^{+}CD1d-PBS57^{+}) in the thymus (top) and spleen (bottom) of WT and *Tsc1*$^{-/-}$ mice. Left panel, representative flow cytometric plots; Right panel, the absolute number of NKT cells in the thymus and spleen of WT and *Tsc1*$^{-/-}$ mice. (c) Expression of CD62L vs CD44 on splenic CD4^{+} (top) or CD8^{+} T cells (bottom) from WT and *Tsc1*$^{-/-}$ mice. (d) Left panel, Representative flow cytometric plots of thymocytes in WT and *Tsc1*$^{-/-}$ mice. Right panel, the absolute number of CD4^{+} single-positive (CD4SP) and CD8^{+} single-positive (CD8SP) thymocytes. (e) Expression of maturation markers CD62L and CD69 on WT and *Tsc1*$^{-/-}$ CD4SP (top) or CD8SP (bottom) thymocytes. Each symbol represents an individual mouse; Data are shown as means ± s.d and represent at least three independent experiments. . ***$p < 0.001$, NS, no significant difference.

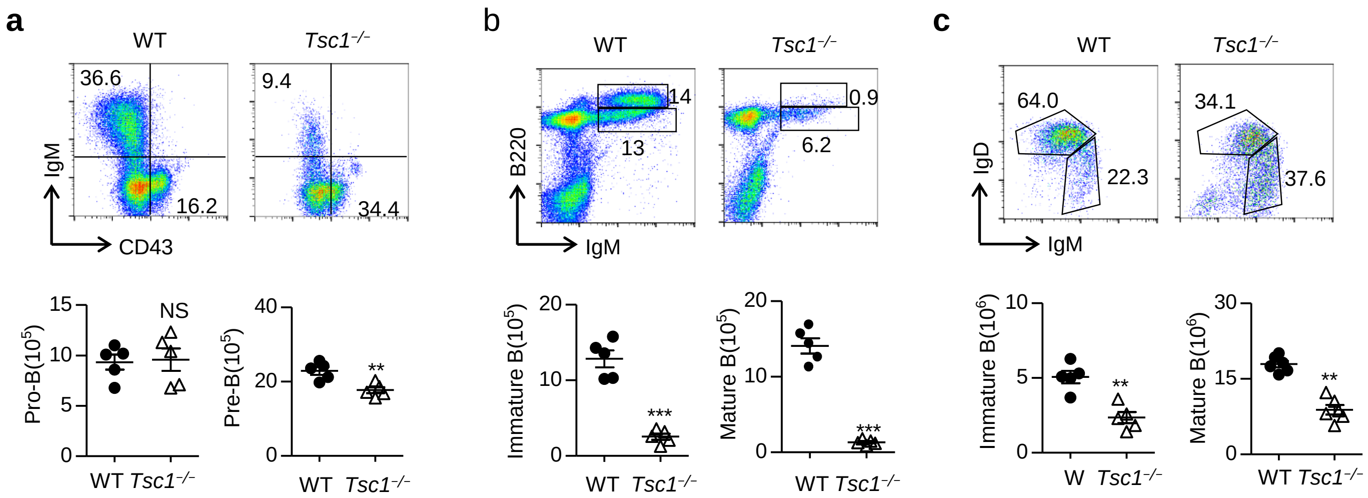

Supplementary Figure 3. Hematopoietic-specific deletion of Tsc1 blocked early pro-B-cell development.

(a) Top, expression of IgM and CD43 of B220+ B cells from WT and *Tsc1*$^{-/-}$ mice. Bottom, the absolute number of indicated cells. (b) Top, expression of IgM and B220 of bone marrow cells from WT and *Tsc1*$^{-/-}$ mice. Bottom, the absolute number of indicated cells. (c) Top, expression of IgM and IgD of splenocytes from WT and *Tsc1*$^{-/-}$ mice. Bottom, the absolute number of indicated cells. Each symbol represents an individual mouse. All data represent at least three independent experiments and calcultated data are shown as means ± s.d. $^{**}p < 0.01$,$^{***}p < 0.001$.

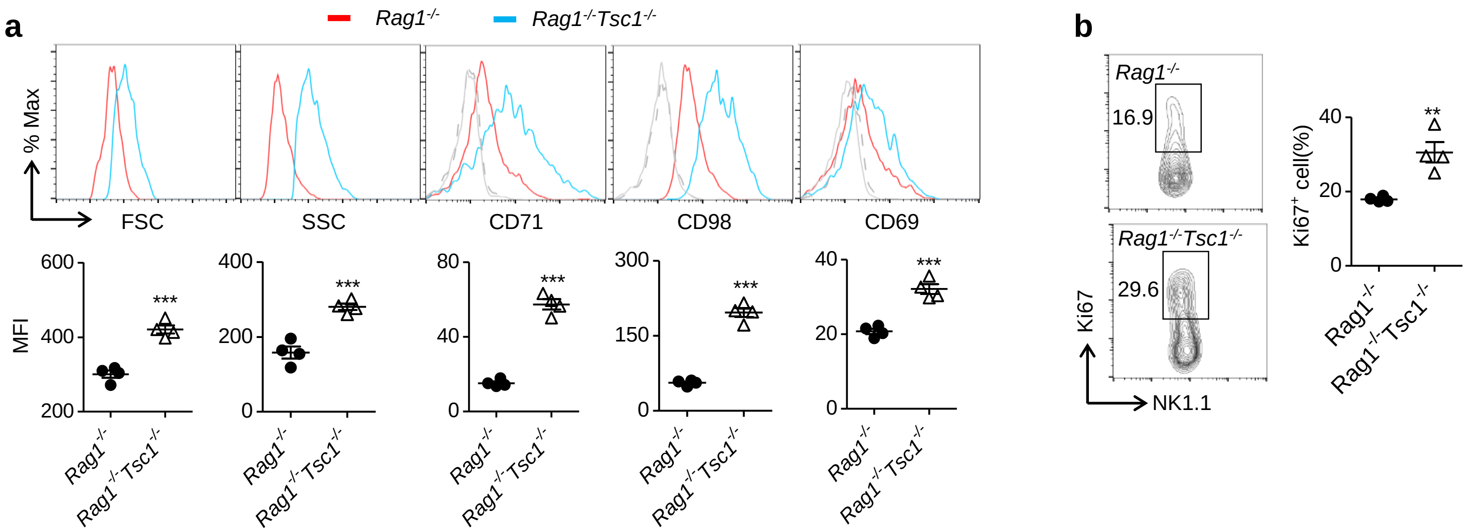

Supplementary Figure 4. The constitutive metabolic activation in the *Tsc1*$^{-/-}$ mice was NK-cell intrinsic.
(a, b) Tsc1-deleted mice were further bred onto *Rag1*$^{-/-}$ mice that lack T, and B cells. The cell size, FSC, SSC, and the expression of CD71, CD98, CD69 (a) and the proliferation marker Ki67 (b) of splenic NK cells from WT and *Tsc1*$^{-/-}$*Rag1*$^{-/-}$ mice were analyzed using flow cytometry. Each symbol represents an individual mouse; Data are shown as means ± s.d and are representative of three independent experiments. . $**p < 0.01$,$***p < 0.001$.

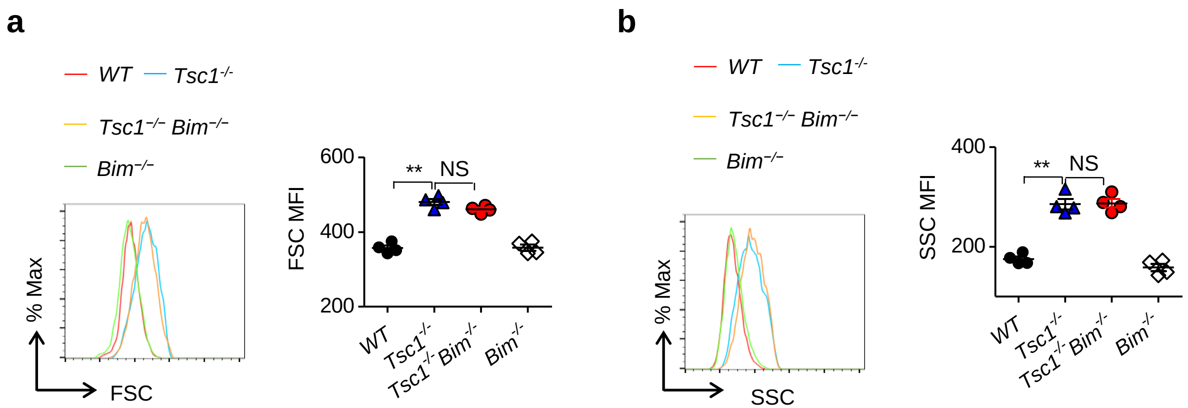

Supplementary Figure 5. The deletion of Bim failed to correct the increased metabolic activation in the *Tsc1*$^{-/-}$ mice.

(a, b) Left panel, representative overlaid histograms showing FSC (cell size) and SSC (cell containing) from the indicated mice. Right panel, MFI of FSC and SSC was calculated. Each symbol represents an individual mouse; Data are shown as means ± s.d and are representative of three independent experiments. . ***p* < 0.01, NS, no significant difference.

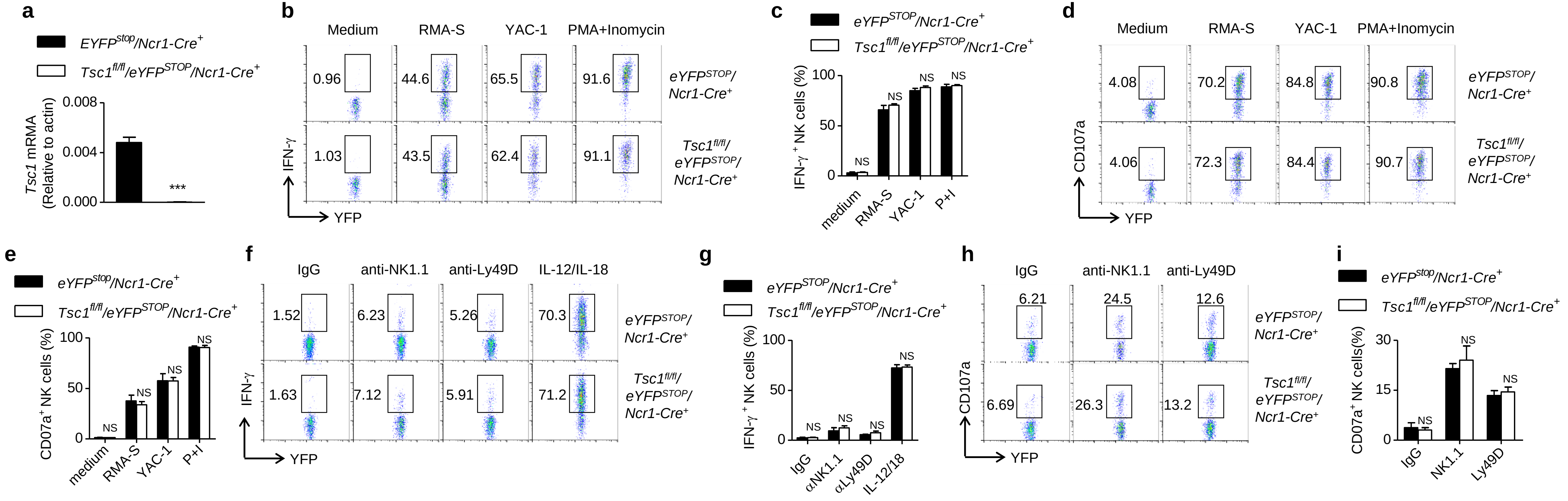

Supplementary Figure 6. NK-cell fate mapping revealed that Tsc1 was not required for effector functions.

(a) Quantitative RT-PCR analysis of *Tsc1* expression in sorted $CD3^{-}NK1.1^{+}YFP^{+}$ cells from indicated mice. (b-i) *RosaEYFP*$^{fl/+}$*Ncr1-Cre*$^{+}$ and *Tsc1*$^{fl/fl}$*Rosa EYFP*$^{fl/+}$*Ncr1-Cre*$^{+}$ mice were injected intraperitoneal with polyI:C (10 mg/mg) and sacrified after 18 hours. Splenic lymphocytes were prepared and co-cultured with an equal number of tumor cell lines, RMA-s and YAC-1, and indicated antibodies; medium alone served as a negative control, and PMA (P) plus ionomycin (I) served as a positive control. Intracellular staining was used for the detection of IFN-γ production by *EYFP*$^{+}$ NK cells (b, f). CD107a expression on *EYFP*$^{+}$ NK cells was chosen as a marker for NK cell degranulation (d, h). The bar graphs show the averaged percentages of IFN-γ^{+} cells (c, g) and CD107a^{+} cells (e, i) of 3-4 experiments. Data represent the mean ±s.d of 3-4 mice per group and are representative of three independent experiments. ***p < 0.001, NS, no significant difference .
